# Remote ischemic postconditioning increased cerebral blood flow and oxygenation assessed by magnetic resonance imaging in newborn piglets after hypoxia-ischemia

**DOI:** 10.3389/fped.2022.933962

**Published:** 2022-09-29

**Authors:** Sigrid Kerrn-Jespersen, Mads Andersen, Kristine Bennedsgaard, Ted Carl Kejlberg Andelius, Michael Pedersen, Kasper Jacobsen Kyng, Tine Brink Henriksen

**Affiliations:** ^1^Department of Paediatrics and Adolescent Medicine, Aarhus University Hospital, Aarhus, Denmark; ^2^Department of Clinical Medicine, Aarhus University Hospital, Aarhus, Denmark; ^3^Comparative Medicine Laboratory, Department of Clinical Medicine, Aarhus University, Aarhus, Denmark

**Keywords:** hypoxic-ischemic encephalopathy, remote ischemic postconditioning, magnetic resonance imaging, arterial spin labeling, blood-oxygen-level-dependent

## Abstract

**Background:**

We have previously investigated neurological outcomes following remote ischemic postconditioning (RIPC) in a newborn piglet model of hypoxic-ischemic encephalopathy. The aim of this study was to further investigate potential mechanisms of neuroprotection by comparing newborn piglets subjected to global hypoxia-ischemia (HI) treated with and without RIPC with regards to measures of cerebral blood flow and oxygenation assessed by functional magnetic resonance imaging.

**Materials and methods:**

A total of 50 piglets were subjected to 45 min global HI and randomized to either no treatment or RIPC treatment. Magnetic resonance imaging was performed 72 h after the HI insult with perfusion-weighted (arterial spin labeling, ASL) and oxygenation-weighted (blood-oxygen-level-dependent, BOLD) sequences in the whole brain, basal ganglia, thalamus, and cortex. Four sham animals received anesthesia and mechanical ventilation only.

**Results:**

Piglets treated with RIPC had higher measures of cerebral blood flow in all regions of interest and the whole brain (mean difference: 2.6 ml/100 g/min, 95% CI: 0.1; 5.2) compared with the untreated controls. They also had higher BOLD values in the basal ganglia and the whole brain (mean difference: 4.2 T2*, 95% CI: 0.4; 7.9). Measures were similar between piglets treated with RIPC and sham animals.

**Conclusion:**

Piglets treated with RIPC had higher measures of cerebral blood flow and oxygenation assessed by magnetic resonance imaging in the whole brain and several regions of interest compared with untreated controls 72 h after the HI insult. Whether this reflects a potential neuroprotective mechanism of RIPC requires further study.

## Introduction

Perinatal hypoxia and ischemia (HI) may lead to neonatal hypoxic-ischemic encephalopathy (HIE). HIE is an acute encephalopathy that occurs in 1–3/1,000 newborns, which is associated with both infant mortality and neurological disabilities (1, 2). Therapeutic hypothermia is the only current neuroprotective treatment of HIE; but with a number needed to treat of seven for an additional beneficial effect, further inexpensive and easily applicable therapies are needed (3). Especially as the incidence of HIE is higher in low- and middle-income countries where therapeutic hypothermia may be difficult to perform correctly and where deleterious effects of the treatment recently have been reported (4, 5). The neuroprotective effects of remote ischemic postconditioning (RIPC) have been investigated in several HI animal models with conflicting results (6–12). We previously found that RIPC improved brain lactate/*N*-acetyl-aspartate (Lac/NAA) ratio in newborn piglets compared with untreated controls by magnetic resonance spectroscopy 72 h after HI (6). The Lac/NAA ratio has been shown to be an early and robust predictor of neurological outcomes in human newborns (13, 14). However, we found no association between RIPC and other markers of brain injury including diffusion-weighted imaging, histopathology, and functional measures. Another HI piglet study has also found that RIPC of 10 min ischemia and reperfusion improved brain Lac/NAA ratio and histological cell death; but only in the white matter (7). A recent review highlighted the need for further studies with focus on the potentially neuroprotective mechanism of RIPC and application of clinically relevant biomarkers for the bridging of results from animal to human studies (15).

Changes in cerebral perfusion and tissue oxygenation may play important roles in the development of neonatal HI brain injury (16). After acute HI causing cell death, reperfusion results in partial recovery with hypoperfusion, reduced cerebral metabolism, and increased tissue oxygenation (17). After 6–24 h, this is followed by secondary injury with hyperperfusion, mitochondrial failure, and delayed cell death (18–21). It has been shown that RIPC may reduce neuronal cell death by upregulating endothelial nitric oxide synthase (eNOS) through activation of the PI3K/Akt pathway (8, 10, 22). eNOS-derived NO release may induce vasodilation in the newborn brain, which may increase cerebral perfusion following the HI insult (23). Thus, improved cerebral perfusion and oxygenation is one of the possible neuroprotective mechanisms of RIPC in neonatal HIE.

Magnetic resonance imaging (MRI) with arterial spin labeling (ASL) is used to assess regional cerebral blood flow (24–27), while blood-oxygen-level-dependent (BOLD) is used to assess local differences in oxygenated and deoxygenated hemoglobin (28, 29). Accordingly, these two MRI modalities may evaluate cerebral hemodynamics and oxygenation. As MRI may be performed non-invasively, it is applicable to human neonates and may therefore be used to evaluate neuroprotective mechanisms in the clinical setting (30). Therefore, we aimed to study the effect of RIPC in newborn piglets subjected to global HI with regards to measures of cerebral blood flow and oxygenation assessed by MRI.

## Materials and methods

The current study presents secondary analyses from our primary study approved by the Danish Animal Experiments Inspectorate (2012–15–2934–00036) (6). Accordingly, this study is in keeping with the 3Rs principle (replacement, reduction, and refinement) of animal research (31). The experiments were conducted at the Department of Clinical Medicine, Aarhus University Hospital, Denmark. The study is reported according to the Animal Research: Reporting of *In Vivo* Experiments (ARRIVE) guidelines (Supplementary material 1; 32).

### Study design

Danish Landrace piglets were included of either sex, less than 25 h of age, and weighing between 800 and 2,500 g. Animals were provided from herds included in a health-monitoring program for slaughter pigs, screened for several pathogens that could affect pigs in a production setting. Fifty-four piglets were included in the study based on sample size calculations previously described (6). Four piglets served as shams and were only exposed to anesthesia and mechanical ventilation. The 50 study piglets were randomized to HI with and without RIPC treatment in blocks of 4–8 animals by sealed envelopes. After 72 h, the piglets underwent MRI including perfusion-weighted (ASL) and oxygenation-weighted (BOLD) sequences.

### Experimental procedures

The HI piglet model and the experimental procedures have been previously described (6, 33). The piglets were anesthetized with a bolus of propofol (5 mg/kg), fentanyl (30 μg/kg), and rocuronium (1 mg/kg) for the endotracheal intubation. The piglets then received continuous intravenous infusion of propofol (5 mg/kg/h) and fentanyl (10 μg/kg/h). Catheters were placed in an umbilical artery for monitoring of blood pressure and blood sampling; and the umbilical vein for drug, glucose, electrolyte, and fluid administration. The piglets were then subjected to a 45 min HI insult. The FiO_2_ was reduced to 4%, while the respiratory rate was reduced to 16 min to mimic the compromised gas exchange in the human newborn insult. This was sustained to produce an amplitude-integrated electroencephalography (aEEG) trace of 7 μV or lower. The FiO_2_ was then gradually increased as long as the aEEG tracing remained below 7 μV. We aimed for hypotension with mean arterial blood pressure (MABP) < 70% of baseline for at least 10 min. Duration of aEEG and MABP suppression during HI have previously been shown to correlate with the severity of brain damage, measured by histological cell death, in newborn piglets (34, 35). Following the HI insult, the piglets were randomized to treatment with and without RIPC. RIPC was performed 1 h after the insult by 5 min occlusion of both hindlimbs followed by 5 min reperfusion in four cycles. An external plastic strip was tightened until complete occlusion of arterial blood flow was verified by Doppler ultrasound, indicating supra-systolic pressure. After these procedures anesthesia, and pain relief were discontinued. When the piglets were stabilized and awake, they were extubated. The piglets were then observed for 3 days while fed every 2 h. To prevent infections, Procaine benzylpenicillin (15.000 IU/kg s.c.) was given daily. Volume replacement and dopamine were used to uphold mean arterial blood pressure, while phenobarbitone was used to treat seizures. After 72 h, the piglets were re-anaesthetized with propofol and fentanyl and subjected to MRI. Immediately after this procedure, the piglets were euthanized with pentobarbital (10 mg/kg).

### Functional magnetic resonance imaging

The piglets were connected to a transportable animal-ventilator. The animals were then transported to the scanner with less than 5 min of transportation time. During the transport, the saturation and heart rate of the animals were monitored by pulse oximeter. During the scanning, only the heart rate was registered by electrocardiography. MRI was conducted using a clinical three Tesla system (Siemens Medical Systems^®^, Munich, Germany). The piglets were placed in a feet-first supine position with the brain surrounded by a multichannel radio frequency knee-coil for data reception. No paralytics were used during the scanning. The sequence protocol was acquired in the following order: (1) T2-weighted spin-echo sequences were used to locate the brain and to prepare subsequent sequences; (2) 3D high-resolution gradient-echo sequences were performed to measure intracerebral volumes in the brain; (3) perfusion-weighted ASL sequences were employed to derive cerebral blood flow measures including a T1-parametric fitted map; (4) BOLD sequences were employed using a gradient-echo echo-planar imaging sequence. All quantitative measures were calculated by Mistar (Apollo Imaging Technology^®^, Melbourne, VIC, Australia) and Siemens Syngo (Siemens Medical Systems^®^, Munich, Germany). ASL was employed as a FAIR sequence using slice-selective-inversion labeling of the targeted slice and independent global inversion labeling, which covers the whole brain. ASL was conducted with repetition time = 3,200 ms, echo time = 25.6 ms, slice thickness = 5 mm, field of view = 14 cm × 14 cm, and slice numbers = 6. Analysis of BOLD data (T2*) was performed from pixel-by-pixel analysis using a non-linear least-squares fit to the logarithmic magnitude vs. echo time. BOLD was conducted with repetition time = 431 ms, echo time = 3.7–29.4 ms, slice thickness = 4 mm, field of view = 11.8 cm × 11.8 cm, and slice numbers = 8. Analyses were performed in the whole brain and regions of interests including the basal ganglia, thalamus, and cortex (Supplementary material 2; 36–38). Whenever image quality allowed, analysis was performed bilaterally in the selected regions. The same researcher drew all regions of interest to reduce interobserver bias and data were managed and analyzed blinded to treatment.

### Statistical methods

Differences were investigated between piglets subjected to HI treated with and without RIPC. Continuous data were analyzed by Student’s *t*-test or Mann-Whitney test and presented as either mean values and standard deviations or median values and interquartile ranges. Categorical data were analyzed by Chi-square test and presented with numbers and percentages. Differences between groups are presented with mean differences (MD) and 95% confidence intervals (CI). Differences in HI insult severity between groups and how they affected the association between treatment and outcome were investigated by Pearson’s correlation and multiple linear regression. A two-sided *p*-value below 0.05 was considered statistically significant. The statistical analyses were performed with GraphPad Prism (GraphPad Prism version 8.4.2 for macOS, GraphPad Software, La Jolla, CA, USA) and Stata 17 software (StataCorp. 2021. Stata Statistical Software: Release 17. College Station, TX: StataCorp LLC). Sham animals were included as a proof-of-concept, i.e., to qualitatively validate that animals subjected to HI differed by MRI measures compared with healthy animals. Sham animals were therefore not included in the statistical analyses.

## Results

### Baseline data

**FIGURE 1:**
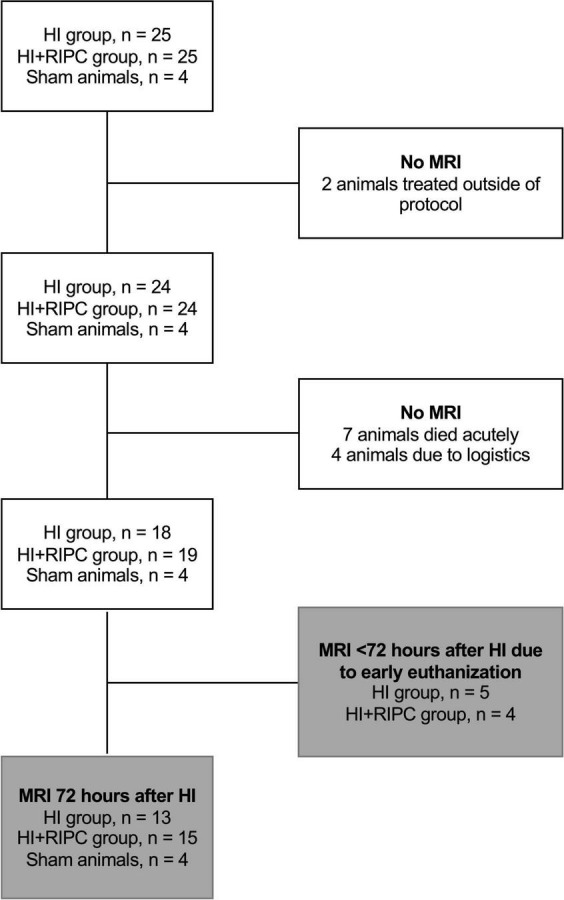
Flowchart of sham animals and animals exposed to hypoxia-ischemia (HI) randomized to no treatment (HI group) or remote ischemic postconditioning (HI + RIPC group) undergoing magnetic resonance imaging (MRI).

Magnetic resonance imaging measurements were available for 18 piglets subjected to HI alone and 19 piglets also treated with RIPC (6). A flowchart of animals undergoing MRI is provided in Figure 1. A total of five piglets subjected to HI alone and four piglets treated with RIPC were euthanized before the 72 h MRI assessment due to respiratory problems including lack of spontaneous breathing after resuscitation; but were still able to be scanned prior to administration of pentobarbital. For piglets subjected to HI alone; one piglet was euthanized between 0 and 24 h, two piglets were euthanized between 24 and 48 h, and two piglets were euthanized between 48 and 72 h. For piglets also treated with RIPC; one piglet was euthanized between 24 and 48 h and three piglets were euthanized between 48 and 72 h. Observed vital signs remained stable during the transport and scanning of the animals. For piglets surviving 72 h; similar values were observed between the two HI groups concerning heart rate, MABP, pCO_2_, pH, and standard base excess. However, *P*-glucose was higher in piglets treated with RIPC at the end of HI and 3 h post HI, while P-lactate was lower at the 72 h MRI assessment (Table 1). With regards to insult severity, piglets treated with RIPC had shorter duration of MABP < 70% of baseline during the HI insult, while the duration of aEEG < 7 μV was similar between groups (Table 1). Similar associations were found when looking at all piglets regardless of the timing of their MRI (Supplementary material 3). A total of five piglets subjected to HI alone and two piglets also treated with RIPC were administered inotropes during the study, while only one piglet subjected to HI alone was treated with phenobarbitone. No animals received inotropes or phenobarbitone during scanning. No signs of limb tissue injury were observed following RIPC.

**TABLE 1:** Descriptive data of sham animals and animals exposed to hypoxia-ischemia randomized to no treatment (HI group) or remote ischemic postconditioning (HI + RIPC group) surviving until 72 h.

	Shams (*n* = 4)	HI (*n* = 13)	HI + RIPC (*n* = 15)	*P*-value
**Characteristics**				
Weight, g	1,913 (452)	1,519 (301)	1,676 (255)	0.15
Age, h	19 (1.1)	20 (1.7)	22 (2.0)	0.06
Females	2 (50%)	6 (46%)	7 (47%)	0.98
**Baseline**				
Heart rate, bpm	151 (9.9)	151 (22.4)	141 (13.7)	0.18
Mean arterial BP, mmHg	No data	52 (5.7)	49 (5.0)	0.26
Arterial pCO_2_, kPa	4.8 (0.5)	5.1 (0.6)	5.0 (0.8)	0.87
pH	7.5 (0.07)	7.5 (0.04)	7.5 (0.08)	0.72
Standard base excess, mM	6.8 (3.0)	8.3 (1.8)	7.1 (2.2)	0.11
Glucose, mM	5.6 (4.7–6.0)	5.5 (4.3–6.8)	6.3 (5.7–7.5)	0.23
Lactate, mM	1.6 (1.3–1.8)	1.8 (1.5–2.0)	1.8 (1.4–2.5)	0.68
**Insult severity**				
aEEG < 7 μV, min		41 (37–42)	41 (31–43)	0.91
MAPB < 70% of baseline, min		11.8 (7.8)	4.1 (6.8)	**0.02**
**End-hypoxia**				
Heart rate, bpm		191 (35.4)	194 (38.3)	0.85
Mean arterial BP, mmHg		52 (19.5)	59 (18.4)	0.40
Arterial pCO_2_, kPa		5.7 (5.5–6.6)	6.1 (5.5–7.4)	0.50
pH		7.0 (0.18)	7.0 (0.17)	0.68
Standard base excess, mM		–16.4 (6.0)	–16.5 (6.3)	0.98
Glucose, mM		10.2 (4.0)	13.9 (4.7)	**0.04**
Lactate, mM		16.6 (3.4)	16.0 (4.7)	0.72
**3 h after hypoxia-ischemia**				
Heart rate, bpm		165 (19.2)	171 (16.2)	0.41
Mean arterial BP, mmHg		47.5 (5.9)	49.1 (6.6)	0.53
Arterial pCO2, kPa		4.7 (0.4)	4.6 (0.5)	0.66
pH		7.5 (0.04)	7.5 (0.07)	0.54
Standard base excess, mM		5.5 (3.7–7.0)	3.2 (1.9–5.9)	0.19
Glucose, mM		5.9 (5.3–7.4)	8.3 (6.5–11.0)	**0.04**
Lactate, mM		2.3 (1.7–3.2)	3.5 (2.2–5.0)	0.07
**At the 72 h assessment**				
Heart rate, bpm	157 (7.9)	136 (20.1)	145 (21.0)	0.24
Temperature, ^°^C	38.7 (0.1)	38.4 (0.6)	38.2 (0.5)	0.47
Arterial pCO2, kPa	4.9 (4.5–5.4)	5.9 (5.4–6.3)	5.4 (5.1–6.8)	0.58
pH	7.5 (0.08)	7.4 (0.09)	7.4 (0.11)	0.18
Standard base excess, mM	6.3 (2.6)	1.4 (3.9)	1.3 (4.1)	0.95
Glucose, mM	5.6 (5.5–5.6)	5.6 (5.0–6.1)	5.6 (5.3–5.9)	0.92
Lactate, mM	1.2 (0.2)	3.8 (1.2)	2.3 (1.1)	**0.01**

We compared the HI and HI + RIPC group. Normally distributed data were analysed by Student’s t-tests and presented as means (standard deviation), while non-normally distributed data were analysed by Mann-Whitney tests and presented as medians (interquartile range). Categorial data were analysed by chi-square tests and presented as n (%). Bold values indicate statistically significant difference.

### Cerebral blood flow by arterial spin labeling perfusion at 72 h

**FIGURE 2:**
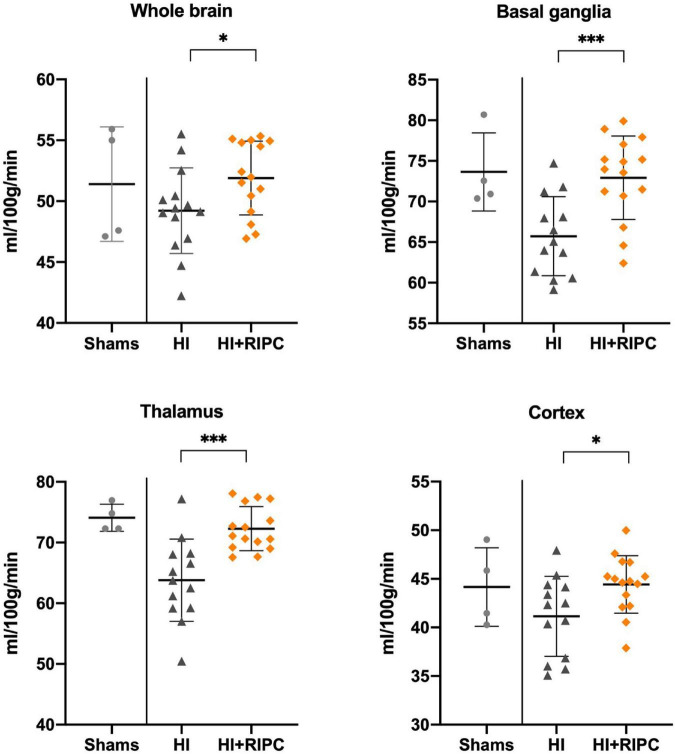
Cerebral blood flow in sham animals and animals subjected to hypoxia-ischemia randomized to no treatment (HI group) or remote ischemic postconditioning (HI + RIPC group) at 72 h. Cerebral blood flow assessed by arterial spin labeling functional magnetic resonance imaging. Data were analysed by Student’s *t*-test and presented with mean values and standard deviations. *P*-values are denoted by: *(<0.05); ^**^(<0.01); ^***^(<0.001).

Arterial spin labeling values between groups are presented in Figure 2. Piglets treated with RIPC had higher measures of cerebral blood flow compared with piglets subjected to HI alone with a statistically significant difference in both the whole brain (MD: 2.6 ml/100 g/min, 95% CI: 0.1; 5.2), basal ganglia (MD: 7.1 ml/100 g/min, 95% CI: 3.3; 11.1), thalamus (MD: 8.5 ml/100 g/min, 95% CI: 4.5; 12.4), and cortex (MD: 3.3 ml/100 g/min, 95% CI: 0.5; 6.1). No differences were found between piglets treated with RIPC and sham animals (whole brain MD: 0.5 ml/100 g/min), while piglets subjected to HI alone appeared to have lower measures of cerebral blood flow compared to sham animals (whole brain MD: –2.2 ml/100 g/min).

### Tissue oxygenation by blood-oxygen-level-dependent imaging at 72 h

**FIGURE 3:**
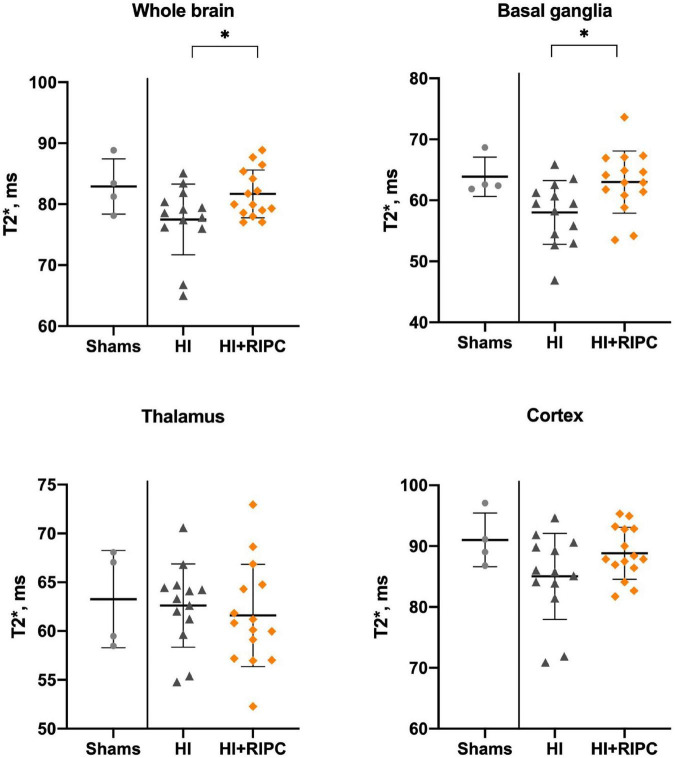
Blood-oxygen-level-dependent imaging in sham animals and animals subjected to hypoxia-ischemia randomized to no treatment (HI group) or remote ischemic postconditioning (HI + RIPC group) at 72 h. T2* signals assessed by functional magnetic resonance imaging. Data were analysed by Student’s *t*-test and presented with mean values and standard deviations. *P*-values are denoted by: *(<0.05).

Blood-oxygen-level-dependent values between groups are presented in Figure 3. Piglets treated with RIPC had higher BOLD values compared with piglets subjected to HI alone with a statistically significant difference in the whole brain (MD: 4.2 T2*, 95% CI: 0.4; 7.9) and basal ganglia (MD: 5.0 T2*, 95% CI: 1.1; 8.9); but not in the thalamus (MD: –1.0 T2*, 95% CI: –4.8; 2.7) or cortex (MD: 3.8 T2*, 95% CI: –0.7; 8.3). No differences were found between piglets treated with RIPC and sham animals (whole brain MD: –1.2 T2*), while piglets subjected to HI alone appeared to have lower BOLD values compared with sham animals in most brain regions with exception of the thalamus (whole brain MD: –5.4 T2*).

### Deceased and surviving piglets

When comparing HI piglets that had an early MRI due to deterioration with those who survived until the 72 h MRI assessment, no statistically significant differences were seen in any MRI measure in any brain region (Supplementary material 4). When conducting intention-to-treat analyses with inclusion of all animals regardless of MRI timing; the differences in measures of cerebral blood flow remained (whole brain MD: 2.9 ml/100 g/min, 95% CI: 0.1; 5.8), while the statistically significant differences in BOLD values disappeared (whole brain MD: 2.5 T2*, 95% CI: –0.8; 5.9) (Supplementary materials 5, 6).

### Additional analyses

We found no correlation between duration of MABP < 70% of baseline during the HI insult and ASL (r = –0.15, *p* = 0.4) or BOLD values (r = –0.21, *p* = 0.3) in the whole brain (Supplementary material 7). After adjustment for the duration of MABP < 70% of baseline during the HI insult by multiple linear regression; the association between treatment group and cerebral blood flow remained (whole brain coefficient: 3.5 ml/100 g/min, 95% CI: 0.3; 6.7), while the statistically significant differences between treatment group and BOLD values disappeared (whole brain coefficient: 3.4 T2*, 95% CI: –1.6; 8.6).

## Discussion

### Summary of findings

We found that piglets subjected to HI treated with RIPC had higher ASL and BOLD values in the whole brain and several regions of interest compared with piglets subjected to HI alone 72 h following the HI insult. This may indicate that RIPC improves cerebral perfusion and oxygenation following HI. RIPC has been suggested to induce neuroprotection by activation of the PI3K/Akt pathway. In adult rats subjected to global cerebral ischemia/reperfusion, Peng et al. (22) found that RIPC reduced neuronal cell death and improved spatial learning and memory through upregulation of eNOS by activation of the PI3K/Akt pathway. Two studies in rats and piglets have investigated how RIPC affects this pathway in neonatal HI. Zhou et al. (8) found that RIPC reduced infarction size and improved functional outcomes in newborn rats through the PI3K/Akt pathway with inhibition of the pathway limiting the neuroprotective effects of the treatment. Moreover, Rocha-Ferreira et al. (10) found that RIPC increased brain expression of eNOS in newborn piglets compared with untreated controls 48 h after carotid-clamping induced HI. The authors speculated that the potential neuroprotective effect of RIPC may be vasodilation through eNOS-derived NO release from the cerebral endothelial cells. However, none of these studies investigated how RIPC specifically affected cerebral hemodynamics. Our findings of increased cerebral blood flow support the hypothesis that RIPC may induce vasodilation in the newborn brain and thereby improves cerebral perfusion. Our results are also in keeping with both animal and human stroke studies that have found conditioning to improve cerebral blood flow (39–44). A randomized clinical trial has previously reported that preconditioning improved cerebral perfusion in stroke patients with intracranial arterial stenosis up to 300 days after the event (39). Upregulation of eNOS has also been proposed as one of the possible mechanisms within this pathology (45). In our previous primary analyses, we found that RIPC was associated with reduced brain Lac/NAA ratio (6, 13, 14). This could indicate that the improved cerebral perfusion is associated with a seemingly more favorable cerebral metabolism. However, our primary analyses also showed that RIPC failed to affect other markers of brain injury including histopathology and crude measures of functional capability (6). Therapeutic hypothermia has also been shown to affect cerebral blood flow following HI. Gunn et al. (46, 47) found that hypothermia suppressed hyperperfusion during the secondary phase of injury from 6 to 48 h after HI in fetal sheep, while improving cerebral blood flow to baseline levels from 72 h. This is similar to the apparent effects of RIPC shown in this study, as we found comparable cerebral blood flow between piglets treated with RIPC and sham animals at 72 h, while HI alone appeared to reduce these measures. We have previously found that RIPC had no additional effect to therapeutic hypothermia on ASL or BOLD values in piglets 24 h after mild HI (11). BOLD values may be used as markers of tissue oxygenation (28, 29). However, it is important to note that several factors may affect BOLD values including blood oxygenation, oxygen extraction, cerebral blood volume, and cerebral blood flow (48–51). The observed BOLD values may therefore also support the differences in ASL values between piglets treated with and without RIPC. As we found RIPC to increase BOLD values in piglets 72 h after HI, this may indicate that RIPC increased cerebral blood flow and/or that RIPC affected cerebral oxygen utilization (52).

### Strengths and limitations

We present a HI piglet model with evaluation of RIPC on clinically translational biomarkers of cerebral hemodynamics and oxygenation. The piglets have comparable brain development and gross anatomy to human newborns and have previously been shown suitable for evaluating early neuroprotective treatments including therapeutic hypothermia (53–55). Among the advantages of this study are the inclusion of both male and female animals and the global HI insult, which may be more clinically relevant compared to other methods like carotid-clamping. However, the current study also has some limitations. As mentioned, RIPC has been suggested to exert neuroprotection by activation of the PI3K/Akt pathway, which may be activated by endogenous opioids (8). We used fentanyl in all piglets, which may have diminished some of the effects of RIPC. As proof-of-concept, we only included four sham piglets to unveil normal ASL and BOLD values following anesthesia and mechanical ventilation only. We anticipated less variation in our MRI measures of sham animals compared to HI animals and therefore decided that four piglets would be appropriate to illustrate these values. At last, the piglets were only evaluated once at 72 h after HI. We chose this timepoint as it may be the earliest relevant time for MRI investigation in human newborns with HIE (13, 14). However, the MRI should ideally have been conducted at multiple time-points after HI to investigate the developments in cerebral blood flow and oxygenation; especially as these may change according to the different stages of HI brain injury, which may be further affected by treatment (16).

## Conclusion

We found that piglets subjected to global HI treated with RIPC had higher measures of cerebral blood flow and oxygenation compared with untreated controls assessed by MRI at 72 h after the HI insult. Whether this may be part of a neuroprotective mechanism of RIPC requires further study.

## Data availability statement

The original contributions presented in this study are included in the article/Supplementary material, further inquiries can be directed to the corresponding author.

## Ethics statement

The animal study was reviewed and approved by Danish Animal Experiments Inspectorate.

## Author contributions

KK and TH: designed the study. SK-J, MP, KK, KB, and TH: piloted the model, refined the study design, and completed the experiments. MA, MP, TA, and SK-J: performed the data-analyses. MA: drafted the manuscript. All authors had critically reviewed the drafted manuscript, approved the manuscript, and agreed to be accountable for all aspects of the work.

## Abbreviations

aEEG: amplitude-integrated electroencephalography
ASL: arterial spin labeling
BOLD: blood-oxygen-level-dependent
eNOS: endothelial nitric oxide synthase
HI: hypoxia-ischemia
HIE: hypoxic-ischemic encephalopathy
Lac/NAA: lactate/*N*-acetyl-aspartate
MABP: mean arterial blood pressure
MRI: magnetic resonance imaging
RIPC: remote ischemic postconditioning.
