# Supplementary material for: Remote ischemic postconditioning increased cerebral blood flow and oxygenation assessed by magnetic resonance imaging in newborn piglets after hypoxia-ischemia
Source: Front Pediatr. 2022 Sep 29;10:933962. doi: 10.3389/fped.2022.933962 (PMC9559709; doi:10.3389/fped.2022.933962)
Supplement: Supplementary file 1 [file Data_Sheet_1.docx]

Supplementary Material

**Title**

Remote ischemic postconditioning increased cerebral blood flow and oxygenation assessed by magnetic resonance imaging in newborn piglets after hypoxia-ischemia

**Authors**

Sigrid Kerrn-Jespersen^12†^, Mads Andersen^12†^, Kristine Bennedsgaard^12^, Ted Carl Kejlberg Andelius^12^, Michael Pedersen^3^, Kasper Jacobsen Kyng^12^, Tine Brink Henriksen^12^

^†^These authors have contributed equally to this work and share first authorship.

**Affiliations**

^1^Department of Paediatrics and Adolescent Medicine, Aarhus University Hospital, Aarhus Denmark

^2^Department of Clinical Medicine, Aarhus University Hospital, Aarhus, Denmark

^3^Comparative Medicine Lab, Department of Clinical Medicine, Aarhus University, Aarhus, Denmark

**Supplementary 1. Reporting according to The Animal Research: Reporting of *In Vivo* Experiments (ARRIVE) guidelines.**

**Supplementary 2. Regions of interest during the magnetic resonance imaging in sham animals and animals subjected to hypoxia-ischemia treated with and without remote ischemic postconditioning.**

**
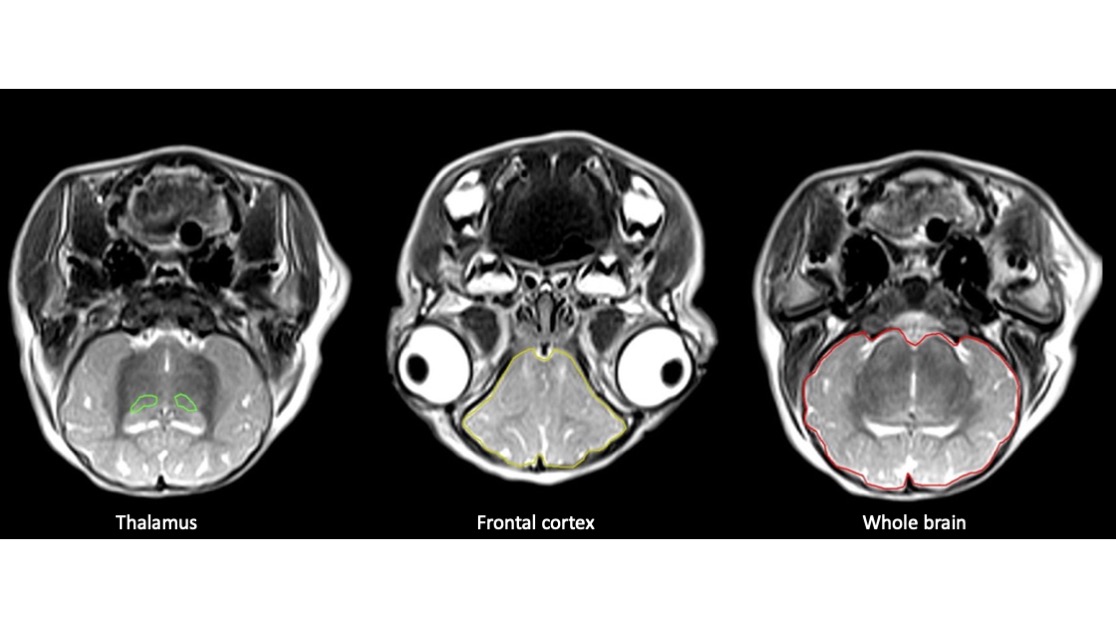
**

The region of interest (voxel placement) for the basal ganglia was based on the following references:

Alderliesten T, de Vries LS, Benders MJ, Koopman C, Groenendaal F. MR imaging and outcome of term neonates with perinatal asphyxia: value of diffusion-weighted MR imaging and ¹H MR spectroscopy. Radiology. 2011;261(1):235-42.

Boichot C, Walker PM, Durand C, Grimaldi M, Chapuis S, Gouyon JB, et al. Term neonate prognoses after perinatal asphyxia: contributions of MR imaging, MR spectroscopy, relaxation times, and apparent diffusion coefficients. Radiology. 2006;239(3):839-48.

**Supplementary 3**. **Descriptive data of all animals exposed to hypoxia-ischemia randomized to no treatment (HI group) or remote ischemic postconditioning (HI+RIPC group).** Normally distributed data were analysed by Student’s t-tests and presented as means (standard deviation), while non-normally distributed data were analysed by Mann-Whitney tests and presented as medians (interquartile range). Categorial data were analysed by chi-square tests and presented as n (%).

|  | **HI**  **(n=18)** | **HI+RIPC**  **(n=19)** | ***p*-value** |
| --- | --- | --- | --- |
| **Characteristics**  Weight, g | 1603 (335) | 1618 (300) | 0.88 |
| Age, h | 20.9 (1.9) | 21.6 (1.9) | 0.25 |
| Females | 7 (39%) | 9 (47%) | 0.74 |
| **Baseline** |  |  |  |
| Heart rate, bpm | 149 (13.5) | 143 (13.5) | 0.25 |
| Mean arterial BP, mmHg | 50.7 (5.6) | 49.2 (5.5) | 0.48 |
| Arterial pCO_2_, kPa | 5.0 (4.8-5.6) | 5.1 (4.6-5.9) | 0.84 |
| pH | 7.5 (0.08) | 7.5 (0.04) | 0.75 |
| Standard base excess, mM | 7.9 (1.7) | 7.8 (2.3) | 0.31 |
| Glucose, mM^a^ | 5.5 (4.4-7.2) | 6.3 (5.4-8.2) | 0.23 |
| Lactate, mM | 1.8 (0.5) | 2.3 (1.1) | 0.17 |
| **Insult severity** |  |  |  |
| aEEG <7 μV, min | 39.5 (33.8-43.0) | 41 (33.3-43.5) | 0.69 |
| MAPB <70% of baseline, min | 11.0 (8.0) | 4.7 (6.7) | **0.03** |
| **End-hypoxia**  Heart rate, bpm  Mean arterial BP, mmHg  Arterial pCO_2_, kPa  pH  Standard base excess, mM  Glucose, mM  Lactate, mM | 188 (34.2)  49 (19.1)  5.9 (5.5-7.7)  7.0 (0.18)  -17.8 (5.8)  10.9 (3.9)  17.5 (3.4) | 197 (37.4)  58 (20.5)  6.1 (5.5-7.4)  7.0 (0.19)  -17.6 (5.9)  13.2 (4.6)  16.6 (4.4) | 0.47  0.23  0.78  0.80  0.92  0.11  0.50 |
| **3 hours after hypoxia-ischemia** |  |  |  |
| Heart rate, bpm | 171 (22.0) | 171 (17.0) | 0.99 |
| Mean arterial BP, mmHg | 46.3 (6.4) | 48.4 (5.9) | 0.37 |
| Arterial pCO2, kPa | 4.7 (0.4) | 4.7 (0.3) | 0.78 |
| pH | 7.5 (7.45-7.54) | 7.5 (7.47-7.53) | 0.90 |
| Standard base excess, mM^c^ | 5.3 (1.8-7.5) | 3.2 (1.9-5.2) | 0.19 |
| Glucose, mM | 6.0 (5.5-7.5) | 8.2 (6.5-10.4) | 0.06 |
| Lactate, mM | 2.9 (1.3) | 3.8 (1.8) | 0.07 |
| **At the 72-hour assessment** |  |  |  |
| Heart rate, bpm | 139 (21.0) | 145 (23.5) | 0.48 |
| Temperature, °C | 38.3 (0.5) | 38.4 (0.5) | 0.20 |
| Arterial pCO2, kPa | 5.0 (0.6) | 5.0 (0.6) | 0.93 |
| pH | 7.4 (0.08) | 7.4 (0.12) | 0.14 |
| Standard base excess, mM | 1.5 (4.1) | 1.1 (3.8) | 0.84 |
| Glucose, mM | 5.6 (5.0-6.2) | 5.6 (5.3-5.9) | 0.93 |
| Lactate, mM | 3.5 (1.3) | 2.3 (1.1) | **0.04** |

**Supplementary 4. Cerebral blood flow (ASL) and blood-oxygen-level-dependent (BOLD) values between piglets subjected to hypoxia-ischemia dying before and surviving until 72 hours.** Data were analysed by Student’s t-test and presented with mean values and standard deviations. No statistically significant differences were found in any region of interest.

**Supplementary 5. Cerebral blood flow in all animals subjected to hypoxia-ischemia randomized to no treatment (HI group) or remote ischemic postconditioning (HI+RIPC group).** Intention-to-treat analyses with inclusion of all animals regardless of deterioration before the 72-hour assessment. Data were analysed by Student’s t-test and presented with mean values and standard deviations.

*p*-values are denoted by: * (<0.05); ** (<0.01); *** (<0.001).

**Supplementary 6. Blood-oxygen-level-dependent imaging in all animals subjected to hypoxia-ischemia randomized to no treatment (HI group) or remote ischemic postconditioning (HI+RIPC group).** Intention-to-treat analyses with inclusion of all animals regardless of deterioration before the 72-hour assessment. Data were analysed by Student’s t-test and presented with mean values and standard deviations. No statistically significant differences were found in any region of interest.

**Supplementary 7. Scatterplot between duration of mean arterial blood pressure (MABP) <70% of baseline during the HI insult and cerebral blood flow (ASL) and blood-oxygen-level-dependent (BOLD) values in the whole brain.**
